# Bone Formation by Sheep Stem Cells in an Ectopic Mouse Model: Comparison of Adipose and Bone Marrow Derived Cells and Identification of Donor-Derived Bone by Antibody Staining

**DOI:** 10.1155/2016/3846971

**Published:** 2016-11-23

**Authors:** Kristian Kjærgaard, Chris H. Dreyer, Nicholas Ditzel, Christina M. Andreasen, Li Chen, Søren P. Sheikh, Søren Overgaard, Ming Ding

**Affiliations:** ^1^Orthopaedic Research Laboratory, Department of Orthopaedic Surgery and Traumatology, Odense University Hospital, Department of Clinical Research, University of Southern Denmark, Sdr. Boulevard 29, 5000 Odense C, Denmark; ^2^Department of Endocrinology and Metabolism, Molecular Endocrinology Laboratory (KMEB), Odense University Hospital, University of Southern Denmark, J. B. Winsløws Vej 25.1, 5000 Odense C, Denmark; ^3^Laboratory of Molecular and Cellular Cardiology, Department of Clinical Biochemistry and Pharmacology, Odense University Hospital, J. B. Winsløws Vej 21.3, 5000 Odense C, Denmark

## Abstract

*Background*. Scaffolds for bone tissue engineering (BTE) can be loaded with stem and progenitor cells (SPC) from different sources to improve osteogenesis. SPC can be found in bone marrow, adipose tissue, and other tissues. Little is known about osteogenic potential of adipose-derived culture expanded, adherent cells (A-CEAC). This study compares* in vivo* osteogenic capacity between A-CEAC and bone marrow derived culture expanded, adherent cells (BM-CEAC).* Method*. A-CEAC and BM-CEAC were isolated from five female sheep and seeded on hydroxyapatite granules prior to subcutaneous implantation in immunodeficient mice. The doses of cells in the implants were 0.5 × 10^6^, 1.0 × 10^6^, or 1.5 × 10^6^ A-CEAC and 0.5 × 10^6^ BM-CEAC, respectively. After eight weeks, bone volume versus total tissue volume (BV/TV) was quantified using histomorphometry. Origin of new bone was assessed using human vimentin (HVIM) antibody staining.* Results*. BM-CEAC yielded significantly higher BV/TV than any A-CEAC group, and differences between A-CEAC groups were not statistically significant. HVIM antibody stain was successfully used to identify sheep cells in this model.* Conclusion*. A-CEAC and BM-CEAC were capable of forming bone, and BM-CEAC yielded significantly higher BV/TV than any A-CEAC group.* In vitro* treatment to enhance osteogenic capacity of A-CEAC is suggested for further research in ovine bone tissue engineering.

## 1. Introduction

In orthopaedic and craniofacial surgery, reconstructing bone defects from trauma, tumours, infection, loose prosthetic components, or surgical procedures presents a medical challenge. Bone defects have traditionally been treated with bone grafts to aid healing, the current gold standard being iliac crest autograft [1]. The autograft is osteoconductive, osteoinductive, and osteogenic but is associated with limited supply and donor site morbidity [1]. Allograft is available as cancellous or cortical graft or demineralized bone matrix. It is osteoconductive and osteoinductive and available in large quantities and is not associated with donor site morbidity. However, allograft is not osteogenic and it carries the risk of disease transmission. The shortcomings of autografts and allografts have motivated the development of bone substitutes [1–3]. Bone substitutes consist of a scaffold, usually containing calcium or other minerals [4]. Scaffolds can be loaded with cytokines and cells to improve osteogenesis and angiogenesis [5, 6].

Osteogenic cells (osteocytes and osteoblasts) originate from SPC that reside in the tissue [7]. MSC are a subset of SPC that are plastic-adherent in culture, can be differentiated into tissue of different lineages, and are positive for markers CD105, CD73, and CD90 and negative for CD45, CD34, CD14 or CD11b, CD79*α* or CD19, and HLA-DR [8]. As MSC can undergo differentiation into tissues unrelated to the tissue in which they originally reside, MSC from one tissue may be useful in another, and MSC for bone substitutes need not to originate from bone and could potentially be harvested from adipose tissue [7, 9]. In contrast to bone marrow, adipose tissue is easier to retrieve and can often be harvested in abundant quantities, A-CEAC show lower levels of senescence and can be expanded to higher passages* in vitro* compared to BM-CEAC [10–12]. This makes adipose tissue an appealing alternative to bone marrow as a source of SPC for bone substitutes, provided that A-CEAC have osteogenic capacity similar to or better than BM-CEAC. Many studies investigate different scaffolds for A-CEAC; however, the number of studies comparing A-CEAC to BM-CEAC with respect to* in vivo* bone formation capacity is very low and varies in species, cells used, characterization method, scaffold, model, and quantitative assessment, thus making direct comparison difficult [13, 14]. BM-CEAC are currently used in clinical settings [15].

The sheep is a frequently used model for orthopaedic research for several reasons: the bone size is large enough to perform complex orthopaedic procedures and for testing medical devices and biomaterials; the lifespan is short enough to perform age-related studies in diseases such as osteoarthritis and osteoporosis [16]; and their bone remodelling is comparable to that of humans [17]. Very little is known about A-CEAC for BTE. To our knowledge, only one study has compared ovine A-CEAC to BM-CEAC with respect to* in vivo* bone formation; furthermore, the comparison was performed in an orthotopic environment [18]. Ovine A-CEAC and BM-CEAC have not yet been compared regarding ectopic bone formation to assess the intrinsic osteogenic capacity, so this would bring new information about ovine A-CEAC.

This study aimed to investigate A-CEAC osteogenesis using BM-CEAC osteogenesis as baseline. The objectives of this study were to use a subcutaneous immunodeficient mouse model [19] to (1) assess the efficacy of A-CEAC on bone formation, (2) compare bone formation between ovine A-CEAC and BM-CEAC, and (3) compare bone formation between different doses of A-CEAC to see if seeding A-CEAC closer improves osteogenesis. Furthermore, we investigate whether a marker for human vimentin (HVIM) can be used to identify ovine cells in implants and thus to reveal the origin of the cells.

We hypothesize that A-CEAC can form new bone in this model, that A-CEAC can form new bone at the same BV/TV as BM-CEAC, that seeding A-CEAC closer improves osteogenesis, and that HVIM can be used to identify sheep cells in histomorphometry.

## 2. Materials and Methods

### 2.1. Study Design

**Figure 1:**
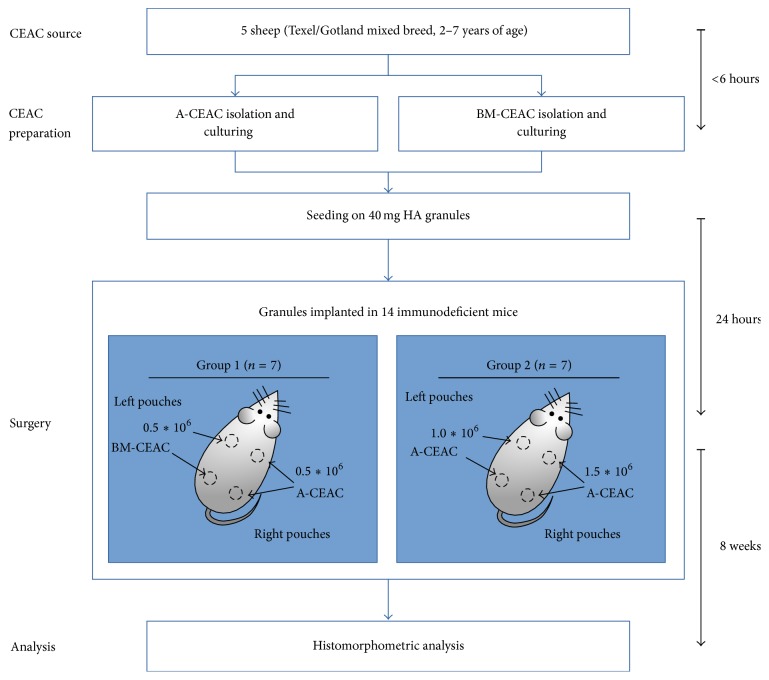
Study design. CEAC from adipose tissue (A-CEAC) and bone marrow (BM-CEAC) was isolated from 5 female sheep, expanded* in vitro,* and seeded onto HA prior to subcutaneous implantation in immunodeficient mice. After 8 weeks, implants were harvested and bone volume versus total tissue volume was assessed by histomorphometry. Each mouse received four implants as previously described [19].

A-CEAC and BM-CEAC were isolated from ovine adipose tissue or iliac crest aspirate, culture expanded, and seeded on hydroxyapatite (HA) granules. Mice were divided into two groups. In mouse group 1, granules seeded with 0.5 × 10^6^ BM-CEAC (denoted BM-CEAC) were implanted in the left pouches, and granules seeded with 0.5 × 10^6^ A-CEAC (denoted A-CEAC1) were implanted in the right pouches. In mouse group 2, granules were seeded with 1.0 × 10^6^ A-CEAC (denoted A-CEAC2) for the left pouches and 1.5 × 10^6^ A-CEAC (denoted A-CEAC3) for the right pouches. Mice and implant groups are shown in Table 1. The workflow is outlined in Figure 1.

### 2.2. Animals

Five female sheep (Texel/Gotland breed, 2–7 years of age) were acquired as donor sheep two months prior to surgery for acclimatization.

Fourteen immunodeficient (NOD.CB17-Prkdc^scid^/J) mice (7-8 weeks of age) were acquired from Charles River, Saint-Germain-sur-l'Arbresle, France, one week before surgery. They had free access to sterilized feed (Altromin 1342 Rat/Mouse, Brogaarden, Lynge, Denmark) and water.

This study was approved by the Danish Animal Experiments Inspectorate (2012-15-2934-00704) and followed national and institutional guidelines.

### 2.3. A-CEAC Isolation and Expansion

Subcutaneous fatty tissue was excised from left lumbar side of sheep. Analgosedation was initialized with midazolam (B. Braun Medical, Frederiksberg, Denmark) 1.0 mg/kg i.v. and maintained with ketamine (Intervet Danmark, Ballerup, Denmark) and midazolam i.v. in an 8 : 1 ratio, and local anaesthesia was achieved using 5 mL lidocaine s.c. (Amgros, Copenhagen, Denmark). A small incision was made through the skin, eight gram fatty tissue was excised, and the incision was closed in layers.

A-CEAC isolation was done as previously described [20]. Briefly, fatty tissue was washed and minced extensively. Tissue was digested with 0.35% Type II collagenase (Worthington, UK) at 37°C for 60 min, washed twice, and filtered through a 100 *μ*m cell strainers to obtain a single cell suspension.

After red blood cell lysis, cells were cultured in DMEM supplemented with 10% foetal bovine serum (FBS; Sigma-Aldrich, Copenhagen, Denmark), 1% penicillin-streptomycin (PS; Sigma-Aldrich), and 4 mM L-Glutamine (Lonza, Copenhagen, Denmark) for two passages and pooled.

### 2.4. BM-CEAC Isolation and Expansion

Bone marrow was aspirated from iliac crest of donor sheep as previously described [21]. The aspirate was density gradient centrifuged using a Histopaque™ gradient (Sigma-Aldrich) to obtain mononuclear cells and cultured in Gibco's Alpha Minimal Essential Medium with 10% FBS, 1% Penicillin-Streptomycin-Glutamine, 1% 1 M HEPES buffer, and 1% 100 mM sodium pyruvate (all Gibco products) for two passages. The cell solutions were cryopreserved at −80°C for one month until scaffold preparation. Upon revival, cells were expanded as described above for one passage and pooled.

### 2.5. Colony-Forming Unit Assay

A colony-forming unit assay (CFU-f) was performed to measure the prevalence of stem and progenitor cells in the initial bone marrow aspirate or adipose tissue sample [7]. One sample from each bone marrow aspirate or adipose tissue stromal vascular fraction was taken for the CFU-f assay just prior to culture expansion. 100,000 cells from bone marrow aspirate or 10,000 cells from adipose stromal vascular fraction were seeded in T80 flasks, medium was changed after 7 days, and CFU-f staining was performed 3 days hereafter. Clusters with at least 50 cells were considered colonies. For bone marrow CFU-f samples, all colonies were counted. Adipose tissue CFU-f samples were counted by random sampling of the assay surface and counted using a grid.

### 2.6. Preparation of Scaffold

The day before mouse surgery, cells were seeded onto scaffold granules in growth medium in cut-off syringes as described previously [19]. Scaffolds were HA granules with a diameter of 1.0–2.5 mm (ENGIPORE, Fin-Ceramica, Faenza, Italy) characterized by a high porosity at up to 90% relative to the total volume.

Before seeding, cells were trypsinized and counted, and the appropriate number of cells were suspended in 300 *μ*L growth medium and loaded onto sterile HA granules as described [19]. The scaffolds were incubated for 24 hours (37°C, 5% CO_2_) before implantation.

### 2.7. Ectopic Mouse Model

Four implants were placed subcutaneously in each mouse according to previous report [19]. On the day of surgery, mice were anaesthetized (100 mg/kg ketamine (Ketaminol 10, Intervet, Ballerup, Denmark), 10 mg/kg xylazine (Rompun Vet, Bayer, Germany), and isoflurane (Baxter, Søborg, Denmark)), and scaffolds were placed in pouches according to Table 1. Incisions were closed and mice were kept separate for 7 days and then housed together in groups of 3-4 until termination of study.

After 8 weeks of observation, mice were euthanized by cervical dislocation, and implants were recovered and placed in 4% neutral buffered formaldehyde for 48 hours.

### 2.8. Histology and Histomorphometry

Implants were decalcified in 12.5% ethylenediaminetetraacetic acid (EDTA; Merck Life Science, Hellerup, Denmark) for ten days and embedded in paraffin. Three serial sections were cut 100, 200, and 300 *μ*m into the implant for a total of nine sections. Sections were deparaffinized, stained with haematoxylin-eosin (HE), blinded, and evaluated in random order by the same researcher. At each sectioning level, the two best sections were analysed and averaged. The volume of bone (BV), fibrous tissue (Fb.V), marrow (Ma.V), and remaining granules (Gr.V) were quantified relative to total tissue volume (TV) using stereological software (newCAST™, Visiopharm, Denmark).

Additional sections from the middle level were stained with HVIM (Thermo Scientific, clone SP20, cat.no RM-9120) to detect ovine cells, as HVIM has shown cross-reaction with ovine cells.

### 2.9. Statistical Analyses and Sample Size Calculation

Statistical analyses were performed using Stata 14 (StataCorp, College Station, Texas, USA). For normality and homoscedasticity, Shapiro-Wilk's test and Levene's test were used, respectively. As our data is heteroscedastic, we used the Welch* t*-test to assess pairwise difference between groups. To reduce the risk of type I errors, significance level was reduced using Bonferroni correction. Differences were considered statistically significant at *p* < 0.008 (corresponding to *p* < 0.05 before Bonferroni correction for pairwise comparison of 4 groups).

Sample size for implants was calculated according to [22]. Accepted risk of error of first and second kinds was set to 5% and 20%, respectively. Minimal relevant difference was set to 70%, and standard deviation was set to 58% based on existing data [21]. This results in *n* = 10.78, and thus 11 implants should be included in each group. Each mouse carries 2 implants from 2 groups, so a total of 6 mice should be included in each group. We included 7 mice in each group due to risk of dropout and implant migration, giving a total of 14 implants per group.

## 3. Results

The average CFU-f count from all sheep was 4.96%  ± 9.52 for stromal vascular fraction and 0.07%  ± 0.02 for bone marrow aspirate.

Some implants migrated and merged with another implant during the observation period and were excluded. Number of remaining implants was 10 in each A-CEAC group and 14 in BM-CEAC group.

**Figure 2:**
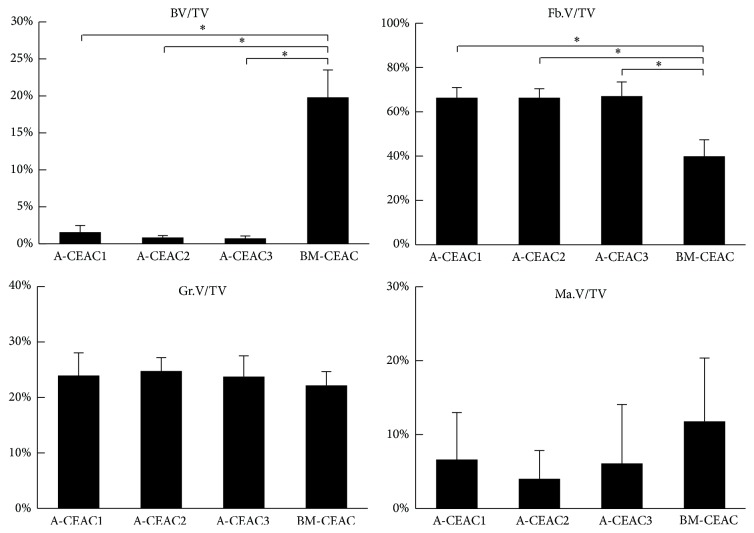
Amount of newly formed bone tissue (BV), fibrous tissue (Fb.V), residual granules (Gr.V), and bone marrow (Ma.V) relative to total tissue volume (TV). A-CEAC1: 0.5 × 10^6^ A-CEAC (*n* = 10); A-CEAC2: 1 × 10^6^ A-CEAC (*n* = 10); A-CEAC3: 1.5 × 10^6^ A-CEAC (*n* = 10); BM-CEAC: 0.5 × 10^6^ BM-CEAC (*n* = 14). ^*∗*^ *p* < 0.002 corresponding to *p* < 0.01 prior to Bonferroni correction.

**Figure 3:**
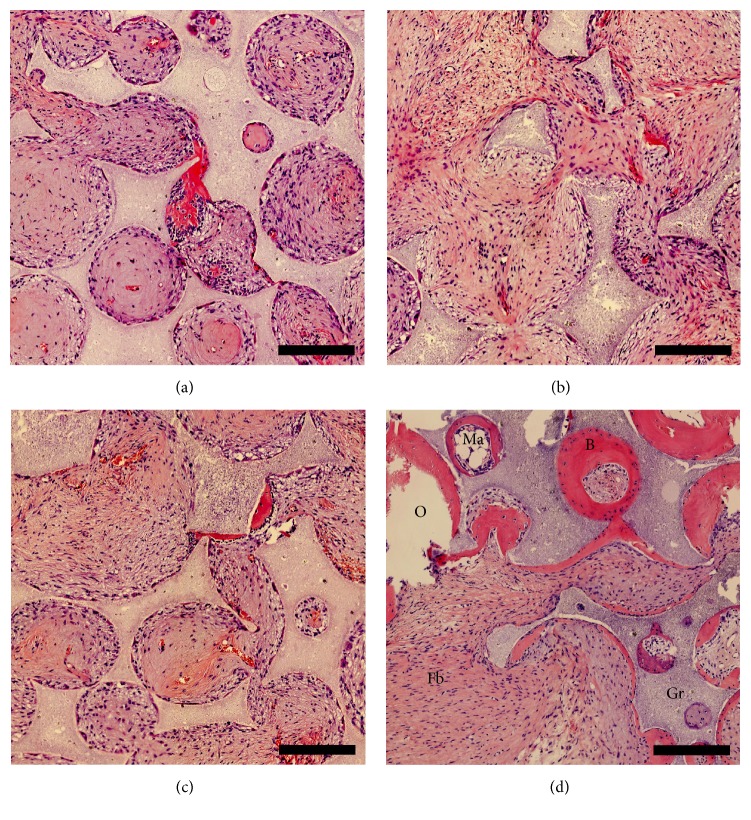
Representative images from A-CEAC1 (a), A-CEAC2 (b), A-CEAC3 (c), and BM-CEAC (d) groups. B: bone; Gr: residual granules; Fb: fibrous tissue; Ma: bone marrow; O: other. Scalebar: 250 *μ*m.

BV/TV, Fb.V/TV, Gr.V/TV, and Ma.V/TV are shown in Figure 2. Significantly higher BV/TV was found in BM-CEAC implants compared to A-CEAC groups. Compared to A-CEAC groups, the BM-CEAC group contained at least 10 times higher BV/TV. Between A-CEAC groups, 0.5 × 10^6^ A-CEAC contained slightly higher BV/TV than the other A-CEAC groups; however, this difference was not statistically significant. In all samples, the present bone tissue was observed adjacent to scaffolds. In samples with lower BV/TV, fibrous tissue dominated the areas adjacent to residual granules. All histological samples except one from A-CEAC3 group showed signs of ossification and vascularization. Gr.V/TV did not differ. BM-CEAC group contained higher Ma.V/TV, both as red and yellow marrow. Representative sample specimens are shown in Figure 3 (A-CEAC1 in Figure 3(a), A-CEAC2 in Figure 3(b), A-CEAC3 in Figure 3(c), and BM-CEAC in Figure 3(d)).

**Figure 4:**
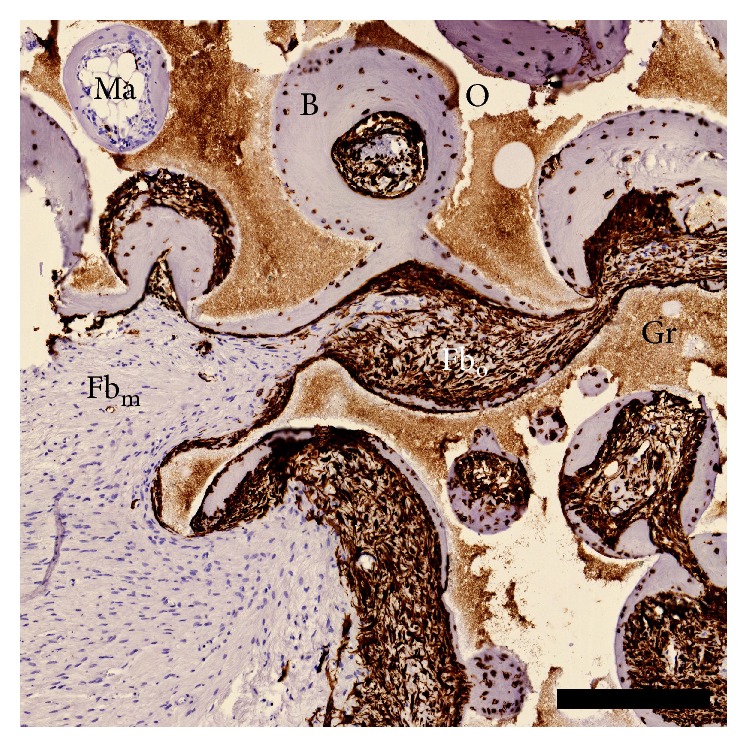
HVIM stain. Tissue stained positive is of ovine origin. All bone tissue in all samples is associated with HVIM+ cells. Fibrous tissue is either HVIM+ or HVIM−. B: bone; Gr: residual granules; Fb_m_: murine fibrous tissue; Fb_o_: ovine fibrous tissue; Ma: bone marrow; O: other. Scalebar: 250 *μ*m.

**Figure 5:**
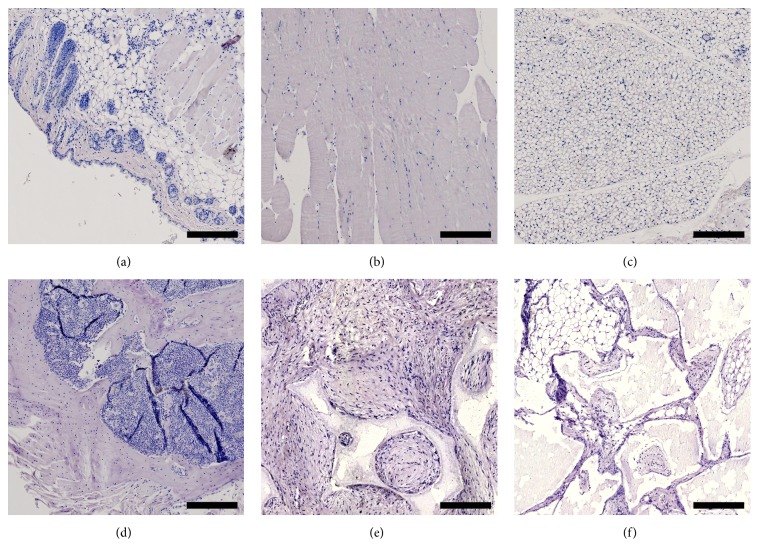
Tissue samples from skin (a), muscle (b), adipose tissue (c), and bone (d), an implant without primary antibody (e), and an implant without cells (f). All samples are HVIM−. Scalebar: 250 *μ*m.

A BM-CEAC sample (corresponding to Figure 3(d)) stained with HVIM is available in Figure 4. Samples from skin (Figure 5(a)), muscle (Figure 5(b)), adipose tissue (Figure 5(c)), and bone (Figure 5(d)) from a control mouse, along with an implant without cells (Figure 5(e)) and an implant with cells (Figure 5(f)) stained without primary antibody, were all negative for HVIM. Negative samples are shown in Figure 5. Residual scaffold was generally connected to HVIM+ tissue (either bone or fibrous tissue), and no HVIM+ tissue was found in areas without residual granules. The implants as whole were encapsulated in HVIM− fibrous tissue, muscle or adipose tissue, with branches reaching into the implant, sometimes separating areas with HVIM+ tissue. All bone tissues were infiltrated with HVIM+ cells, and all osteocytes were HVIM+. Fibrous tissue was either HVIM+ or HVIM−. All areas of marrow were HVIM−. HVIM+ tissue was only found related to residual granules. All HVIM stained specimens were evaluated. We did not distinguish between red and yellow marrow.

## 4. Discussion

We aimed at evaluating A-CEAC and BM-CEAC intrinsic* in vivo* osteogenic capacity and chose not to perform any* in vitro* differentiation prior to implantation. We chose the gold standard for intrinsic osteogenic capacity and the ectopic bone formation model over the orthotopic model to eliminate possible bone formation from residing osteogenic cells [23]. This study demonstrates that it is possible to induce bone formation using A-CEAC and BM-CEAC from sheep, that BM-CEAC form significantly more bone than A-CEAC, that seeding A-CEAC closer does not improve osteogenesis, and additionally that antibody staining against HVIM can be used to identify sheep cells in implants in mice.

The International Society for Cellular Therapy (ISCT) suggested a set of minimal criteria to characterize MSC [8]. These criteria were not met in this study. The cells used in this study were plastic-adherent but were not differentiated to different lineages or sorted by surface markers. Multilineage differentiation has been shown by others using similar isolation techniques [18], and surface markers were not considered relevant, as surface antibodies for the relevant surface antigens have not yet been validated for sheep. The cells used in this study, referenced as A-CEAC and BM-CEAC, were culture expanded and obtained using a similar protocol to obtain MSC [24–26]. Regarding cell characterization criteria in other studies comparing intrinsic osteogenic capacity between A-CEAC and BM-CEAC (see Table 1), all use adherence, most use CD105+, CD90+, CD45−, and CD34−, only a few use differentiation, and none fulfil the total MSC criteria as defined by ISCT [8]. This shows a lack of standardization and qualitative and quantitative assessment in the literature, making direct comparison between studies difficult.

Though BM-CEAC formed significantly more bone than A-CEAC, some bone was present in all A-CEAC implants but one from A-CEAC3 group. Scattered across A-CEAC specimens, several areas characterized by lower cell density and stronger eosin stain than fibrous tissue were found (see centre of Figure 3(a)). These areas were considered fibrous tissue in this analysis and did not qualify as bone tissue. It seems likely that these areas were undergoing mineralization at termination of the study, and a prolonged observation period may yield more bone [27]. However, to what extent a prolonged observation period affects osteogenesis is unknown.

Difference between BV/TV across A-CEAC groups was not significant. A slightly lower BV/TV was observed in A-CEAC2 and A-CEAC3 groups compared to A-CEAC1. An initial investigation into this difference would seek to identify the number of cells attached to granules prior to implantation. In this study, cells were seeded on granules with the assumption that all cells would adhere to the granules, and this assumption has not been verified.

We did not include an empty (cell-free) control group in this study. Our laboratory has previously shown that empty controls contain less than 0.2% BV/TV using the same scaffold and animal [21]. Thus, a paired design with an empty control in each mouse for per-mouse baseline osteogenesis—or an empty group for overall baseline osteogenesis—would have very little effect on the results presented here. Also, osteocytes were HVIM+, and along with the negative panel in Figure 5 all bone tissue was considered of ovine origin.

Some implants migrated and merged with another implant and were excluded, and the number of implants in A-CEAC groups is below our power calculation. While dropout at this magnitude was not expected, we do not believe fewer dropouts would move the results in a less significant direction.

A number of studies have compared A-CEAC to BM-CEAC with respect to bone formation (see Table 2). Ectopic osteogenic capacity has been compared in 2 studies. Brocher et al. compared human A-CEAC to BM-CEAC on tricalcium phosphate subcutaneously in immunodeficient mice for 8 or 12 weeks. They observed new bone more frequently in BM-CEAC specimens than A-CEAC specimens (significance not described), and they observed new bone in A-CEAC specimens more frequently after 12 weeks than after 8 weeks [27]. Hayashi et al. compared rat A-CEAC to BM-CEAC on HA disks subcutaneously for 6 weeks and found that BM-CEAC formed significantly more bone than A-CEAC assessed on *μ*CT [28]. Assessed on histology, Hayashi et al. also observed new bone in all BM-CEAC specimens and none in A-CEAC specimens. These two studies utilize similar scaffolds and a subcutaneous environment comparable to the current study, and both of these studies and the current study show more bone formation from BM-CEAC than from A-CEAC.

Orthotopic osteogenic capacity has also been compared in a few studies. Jo et al. created a segmental femur defect in rats and seeded human A-CEAC or BM-CEAC on an HA/TCP cylinder for implantation [29]. After 12 weeks, significantly higher BV/TV was observed with BM-CEAC compared to A-CEAC assessed on *μ*CT. Conventional X-ray revealed no significant difference. Kang et al. created a segmental radius defect in dogs and compared A-CEAC and BM-CEAC on TCP scaffold for 20 weeks [31]. Neither conventional X-ray nor histomorphometry showed significant difference between groups. Niemeyer et al. created a segmental tibia defect in sheep and compared A-CEAC to BM-CEAC on mineralized collagen scaffold for 26 weeks [18]. Assessed by both conventional X-ray and histomorphometry, A-CEAC were inferior to BM-CEAC on BV/TV. These three segmental defect model studies all show that A-CEAC may be inferior to BM-CEAC when comparing BV/TV. Miyazaki et al. created a spinal fusion model in rats using human A-CEAC or BM-CEAC on a collagen sponge [33]. After 8 weeks, *μ*CT did not reveal a significant difference between A-CEAC and BM-CEAC groups. Wen et al. used human A-CEAC or BM-CEAC on collagen gel in a calvarial defect in rats [32]. After 8 weeks, no significant difference was found between A-CEAC and BM-CEAC groups assessed by conventional X-ray. Taken together, some evidence announce BM-CEAC superior to A-CEAC regarding bone formation capacity, while others show insignificant differences.

Compared to BM-CEAC, A-CEAC may be inferior in bone formation due to (1) stronger commitment to nonbone forming cell lineages or (2) inferior neovascularization properties. Stronger commitment to bone forming cell lineages could be achieved through* in vitro* differentiation, prolonged culturing on scaffold prior to implantation, and transfection or by using cytokine releasing scaffolds. Using a scaffold with functional groups resembling native extracellular matrix may provide a solution to both problems [34]. To clarify whether sheep A-CEAC can be applied in bone substitutes, further investigations in the enhancement of the osteogenic capacity of A-CEAC are needed.

These results do not translate to a clinical setting for several reasons. This study was designed to answer a research question related to basic science, and thus both species and sample size need to be changed to make it translatable to humans. Some differences in osteogenic capacity between human and rhesus monkeys have been shown, and differences between human and sheep have, to our knowledge, not yet been investigated [35].

## 5. Conclusion

A-CEAC seeded on HA granules yielded significantly lower BV/TV than BM-CEAC after eight weeks subcutaneously in immunodeficient mice. This may be due to stronger commitment of A-CEAC to nonbone forming cell lines than BM-CEAC. A-CEAC at a concentration of 0.5 × 10^6^ cells yielded slightly higher BV/TV than A-CEAC at 1.0 × 10^6^ or 1.5 × 10^6^, though this difference was not statistically significant. Human vimentin antibody stain was successfully used to identify ovine cells in our implants. As such, ovine cells do not need pretreatment with labelling techniques for identification in implants in this model. Both A-CEAC and BM-CEAC were capable of forming bone, and bone tissue was found in all histology samples but one from 1.5 × 10^6^ A-CEAC group. Though A-CEAC were capable of forming new bone, some* in vitro* treatment to enhance osteogenic capacity is suggested for further research in ovine bone tissue engineering.

## Figures and Tables

**Table 1:** Animal and implant groups. Cells were seeded on 40 mg HA scaffold as previously described [19].

Mouse group	Pouch	mg HA granules	Cells	Denoted
1 (*n* = 7)	Upper left	40	0.5 × 10^6^ BM-CEAC	BM-CEAC
	Lower left	40	0.5 × 10^6^ BM-CEAC	BM-CEAC
	Upper right	40	0.5 × 10^6^ A-CEAC	A-CEAC1
	Lower right	40	0.5 × 10^6^ A-CEAC	A-CEAC1

2 (*n* = 7)	Upper left	40	1.0 × 10^6^ A-CEAC	A-CEAC2
	Lower left	40	1.0 × 10^6^ A-CEAC	A-CEAC2
	Upper right	40	1.5 × 10^6^ A-CEAC	A-CEAC3
	Lower right	40	1.5 × 10^6^ A-CEAC	A-CEAC3

**Table 2:** Studies comparing A-CEAC and BM-CEAC without osteogenic gene transfection or differentiation prior to implantation. Only data related to bone formation is shown. All studies used CEAC from 2nd to 5th passages. *p* values shown when available. Adapted with modifications from Liao and Chen [13].

Author	Cell origin	Cell characterization	Animal model	Scaffold	Observation time	Analytic method	Outcome
Brocher et al. [27]	Human	Adherence, CD105+, CD73+, CD90+, CD34−, CD45−	Subcutaneous in mice	TCP granules	8 weeks	Histology (new bone observed in specimens)	A-CEAC: 0/22 BM-CEAC: 11/14
					12 weeks		A-CEAC 2/6 BM-CEAC 5/6

Hayashi et al. [28]	Rat	Adherence, CD90+, CD29+, CD45−	Subcutaneous	HA disk	6 weeks	*μ*CT (bone volume)	A-CEAC^a^ 0.05 mm^3^ ± 0.05 BM-CEAC 6.85 mm^3^ ± 1.89 (*p* < 0.001)
						Histology (new bone observed in specimens)	A-CEAC: none BM-CEAC: all

Jo et al. [29]	Human	Adherence, CD105+, CD73+, CD90+, CD34−, CD45−, HLA-DR-	Segmental femur defect in rat	HA/TCP (60%/40%) cylinder	12 weeks	X-ray (bone formation score [30]*⁠*)	BM-CEAC^b^: 4.00 ± 0.63 A-CEAC^b^: 3.17 ± 0.75 (n.s.)
						CT (BV/TV)	BM-CEAC: 14.2% ± 1.4 A-CEAC: 10.4% ± 1.2 (*p* < 0.05)

Kang et al. [31]	Dog	Adherence, CD73+, CD90+, CD44+, CD34−, CD45−, CD14−	Segmental radius defect	TCP/PLGC	20 weeks	X-ray (radiographic healing)	BM-CEAC^c^: 7/8 A-CEAC^c^: 6/8
						Histomorphometry (BV/TV)	A-CEAC: 33.90% ± 4.31 BM-CEAC: 33.56% ± 8.09 (n.s.)

Liao and Chen [13]	Human	Adherence	Spinal fusion in rats	Collagen sponge	8 weeks	*μ*CT (BV/TV)	Insignificant difference between A-CEAC^d^ and BM-CEAC^d^
						Histology (description)	No bridging observed in A-CEAC or BM-CEAC groups

Niemeyer et al. [18]	Sheep	Adherence, differentiation into bone/cartilage/fat	Segmental tibia defect	Mineralized collagen type I	26 weeks	X-ray (relative bone area in defect)	A-CEAC inferior to BM-CEAC (*p* < 0.05)^e^
						Histomorphometry (BV/TV)	A-CEAC inferior to BM-CEAC (*p* < 0.01)

Wen et al. [32]	Human	Adherence CD105+, CD90+, CD29+, CD44+, CD34−, differentiation into bone/fat	Calvarial defect in rats	Collagen gel	8 weeks	X-ray (average grey level)	Insignificant difference between A-CEAC and BM-CEAC

This study	Sheep	Adherence	Subcutaneous in mice	HA granules	8 weeks	Histomorphometry (BV/TV)	A-CEAC: 1.78% ± 0.91 BM-CEAC: 20.87% ± 3.70 (*p* < 0.0017)^f^

^a^A-CEAC from pellet shown here, ^b^undifferentiated groups shown here, ^c^time to healing was not significant, ^d^transfected with LacZ reporter gene; data available only in figure, ^e^data available only in figure, and ^f^ *p* value corresponds to *p* < 0.01 prior to Bonferroni correction.
